# The effects of working memory training on attention deficit, adaptive and non-adaptive cognitive emotion regulation of Chinese children with Attention Deficit/Hyperactivity Disorder (ADHD)

**DOI:** 10.1186/s40359-024-01539-6

**Published:** 2024-02-05

**Authors:** Dan Zhao, Jifang Zhang

**Affiliations:** 1 School of Philosophy and Sociology, Jilin University, Changchun, Jilin, 130012 China; 2 School of Education, Changchun Normal University, Changchun, Jilin, 130032 China; 3 The 964th Hospital No.4799 XianRoad, Lvyuan District, Changchun, 130012 China

**Keywords:** Working memory training, Attention deficit, Adaptive emotion regulation, Cognitive emotion regulation, Non-adaptive cognitive emotion regulation, Attention Deficit/Hyperactivity Disorder (ADHD)

## Abstract

**Background:**

Attention Deficit/Hyperactivity Disorder (ADHD) poses cognitive and emotional challenges for Chinese children. This study addresses the potential benefits of Working Memory Training for ADHD-affected children. Understanding its impact on Attention, cognitive regulation, and emotional responses is crucial for tailored interventions in the Chinese context. The Trial Registration Number (TRN) for this study is [TRN-2023-123,456], and it was officially registered on July 15, 2023, by Changchun Normal University.

**Objectives:**

This study investigated how Working Memory training influences Attention, adaptive cognitive regulation, and non-adaptive cognitive emotion regulation in Chinese children with ADHD. It also assessed changes in attentional focus, improvements in adaptive cognitive regulation, and alterations in non-adaptive cognitive emotion regulation strategies.

**Methodology:**

This quasi-experimental study aimed to assess the impact of working memory training on Chinese children with ADHD. Using pretest-posttest measures, 120 female students underwent Cogmed software training, targeting attention deficits and cognitive emotion regulation. Three reliable instruments measured outcomes. The procedure involved informed consent, questionnaires, 25 training sessions, and a two-month follow-up. Statistical analyses, including repeated measures ANOVA, assessed training effects.

**Results:**

ANOVA revealed a significant impact of Working memory training on attention deficit. Repeated measures ANOVA for cognitive emotion regulation indicated positive changes in adaptive and non-adaptive strategies over time, with sustained improvements in self-blame, rumination, catastrophizing, and blaming others. Bonferroni follow-up tests showed significant differences between pre-test, post-test, and follow-up, favoring the post-test and follow-up tests.

**Conclusions:**

In summary, this research sheds light on the positive impact of memory training on Attention and cognitive emotion regulation in children with ADHD. The study underscores the potential of working memory interventions, particularly software-focused approaches, in enhancing attention levels and improving cognitive emotion regulation. The findings align with existing literature emphasizing the role of working memory deficits in ADHD.

**Implications:**

Practically, incorporating memory training interventions into educational settings emerges as a viable strategy to support children with ADHD. This includes integrating memory training programs into both classroom activities and home-based interventions. Additionally, sustained implementation and long-term follow-up assessments are crucial for maximizing the effectiveness of memory training interventions. Tailoring interventions to specific ADHD subtypes and seamlessly integrating memory training activities into daily routines offer practical and personalized solutions for managing ADHD symptoms in diverse settings.

## Introduction

Working memory is precisely characterized as a cognitive system that temporarily holds limited information amid competing cognitive activities [1–8]. Correspondingly, the capacity of working memory represents the boundary of an individual’s ability to retain pertinent information amidst cognitive distractions, influenced by the competition for limited cognitive resources [2]. Previous research has established that working memory capacity is not specific to a particular domain and is connected to various cognitive activities. Importantly, individuals with high working memory capacity outperform those with low capacity in tasks requiring the suppression of distracting information. For instance, in the Stroop task, participants with higher working memory capacity find it easier to disregard the automatic response to color words, demonstrating the significance of understanding working memory for cognitive development and processing. Furthermore, working memory deficits are a plausible explanation for various developmental cognitive disorders.

Emotional regulation, a higher-level cognitive ability, involves conscious efforts to regulate and override emotional states, forming a system where individuals manage and control their emotionally charged states [3–11]. Recognizing the impact of emotional regulation is crucial, as chronic deficits negatively affect physical well-being and contribute to psychopathology. Given that emotion regulation and emotional working memory both fall within the domain of social cognition, it is imperative to investigate their relationship, especially considering the scarcity of research in this area. Additionally, exploring the effectiveness of working memory training on emotion regulation is essential.

Working memory training has also been found useful for enhancing the Attention and hyperactivity of Children with Attention Deficit/Hyperactivity Disorder (ADHD). ADHD is a neurodevelopmental disorder persisting across the lifespan, characterized by core symptoms of inattention, hyperactivity, and impulsivity [12]. The prevalence of ADHD has risen over time, with approximately 9.4% of children aged 2 to 17 (6.1 million children) diagnosed with the disorder [13]. Gender-specific prevalence rates in 2010 indicated higher ADHD rates in males compared to females across various ethnicities [14]. The significant impairments associated with ADHD, affecting academic performance, social interactions, and overall well-being, underscore the need for effective interventions [15].

Cognitive alterations, including deficits in executive functions, are characteristic of ADHD [16]. Central to ADHD are impairments in verbal and spatial working memory (WM), planning, Attention, vigilance, temporal processing, inhibition, emotional regulation, preference for immediate rewards, and overall decision making [2, 17–20]. While effective in the short/medium term, traditional pharmacological interventions present limitations such as adverse effects, uncertainty about long-term benefits, poor adherence, and negative attitudes [2, 17–19].

In recent years, working memory (WM) training has emerged as a potential non-pharmacological intervention for ADHD [21]. WM training involves adaptive brain games targeting Attention, concentration, verbal and visual WM, processing speed, and inhibition [19, 20, 22]. Adaptive models, adjusting difficulty based on performance, have shown gains in cognitive and WM tasks after 20 hours of intervention, with maintained improvements observed over 6 months [23, 24]. Notably, n-back tasks have effectively decreased WM deficits over time [25, 26]. The potential of WM training extends to academic skills, with studies demonstrating improvements in reading and math [27, 28].

While promising, the effectiveness of WM training varies based on factors like duration, baseline performance, supervision, game elements, motivation, and the types of academic skills measured [29–33]. This prompts a closer examination of the main characteristics of WM training and its impact on the quality of life and psychological well-being in individuals with ADHD.

A child must manifest at least 6 9 symptoms, and adolescents must exhibit at least 5 of the 9 symptoms clustered before the 3 subtypes [12]. Previous research has shown that symptoms related to hyperactivity tend to decrease as the child ages, while inattentiveness remains relatively unchanged [13, 14]. Barkley identified the primary characteristic of ADHD as difficulty in behavioral inhibition [15].

Unintentional behavior displayed by children diagnosed with ADHD appears to impact their academic achievement throughout their academic careers. Additionally, these children often encounter challenges with social skills and communication due to frustrations stemming from academic performance [16]. The DSM-V notes a higher rate of ADHD in males than in females, with a ratio of nearly 2:1 in children and 1.6:1 in adults [11].

Diverse theories exist regarding the causes of attention deficit, involving a combination of biologically determined nervous system reactivity, self-regulation failures, and environmental disturbances (17, 18). Students struggling with focus may experience impatience and negative responses from their environment, potentially leading to a perception of social rejection. The persistent experience of rejection at school, combined with the vulnerability of the child/adolescent nervous system, is believed to generate negative perceptions, prompting compensatory behaviors like aggressive comic behavior, feelings of isolation, and depressive states. Establishing and maintaining a supportive and caring home environment is crucial in alleviating such symptoms. This can be particularly challenging if parents themselves display symptoms similar to their child. ADHD indicators are not confined to childhood, and the dynamic nature of this disorder may contribute to its persistence.

Drawing on prior studies, the primary treatments for ADHD have centered on medication, behavioral, cognitive, cognitive-behavioral, and neural interventions (19–24). A literature review spanning from 2000 to 2013 highlighted multimodal treatments for ADHD in children and adolescents. These combination treatments encompass self-control, self-regulation, cognitive, cognitive-behavioral, cognitive play, behavioral play, motor-perceptual rehearsal, functions management training, verbal self-education training, parent education, positive therapy, parent training, constructive parental programs, and stress coping skills training. These studies demonstrated that the mentioned treatments were more effective and had longer-lasting effects on attention deficit, social skills, and behavioral problems in ADHD compared to singular medication or behavioral therapy [17–20, 22, 25, 26].

Current research on working memory has explored its connection to cognitive activities such as language processing, visual attention control, and filtering irrelevant information in the dichotic listening task. Existing findings highlight the significant role of working memory in goal-relevant processing amid irrelevant information and distractions [2]. Current research trends primarily concentrate on enhancing working memory through visual, verbal, auditory, and cross-modal memory tasks. Despite working memory being considered domain-general, research indicates that training yields narrow gains on specific functions. Consequently, ongoing research focuses on developing more effective training programs that target specific cognitive tasks or employ improved methods for better generalization and far-transfer effects.

In emotional regulation research, current trends involve categorizing different emotion regulation strategies specifying how individuals manage unwanted emotions. Three distinct strategies include need-oriented, goal-oriented, and person-oriented emotion regulation. Need-oriented strategies focus on experiencing hedonically rewarding states, while goal-oriented strategies align with achieving specific goals. Person-oriented emotion regulation emphasizes holistic bodily functioning.

However, limited research has explored the relationship between working memory and emotion regulation, Attention, and hyperactivity of children with ADHD. Moreover, there needs to be more investigation into the potential of working memory training involving emotional regulation as an intervention for various mental disorders. This study aims to investigate the effects of WM training on emotion regulation, Attention, and hyperactivity of 8–13 year-old Chinese girls attending primary schools and psychological center in Changchun China. More specifically, it attempts to answer the following research questions:

1. Does working memory training have a significant impact on the attention of children with attention-deficit/hyperactivity disorder?
2. Does working memory training have a significant impact on adaptive/ cognitive emotion regulation of children with attention deficit/hyperactivity disorder?
3. Does working memory training have a significant impact non-adaptive cognitive emotion regulation of children with attention deficit/hyperactivity disorder?

### Working memory and attention of children with ADHD

ADHD is a neurodevelopmental condition characterized by inappropriate inattention, hyperactivity, and impulsivity [12–14]. Children with ADHD exhibit cognitive alterations that can impact mental health, social skills, behavioral performance, and academic achievement, potentially persisting into adulthood [16]. One key affected aspect is working memory (WM), an executive function vital for storing and manipulating information, crucial in everyday functions like Attention, problem-solving, learning, and decision-making [17]. Deficiencies in working memory are commonly observed in ADHD, predicting challenges in academics, impulsive decision-making, and inattention symptoms [2, 15, 16].

Recent interest has emerged among healthcare professionals, parents, and researchers for interventions targeting WM. WM training is linked to cognitive improvements, general life functioning benefits, and enhanced ADHD-related inattention [10–12]. The effectiveness of WM training is believed to involve neuro-plastic changes in the pre-frontal and frontal brain systems responsible for attentional control and higher-level executive functions [16]. However, studies indicate that interventions focusing solely on a single cognitive domain, such as WM, often fail to positively affect other cognitive functions or domains, termed ‘far transfer.’ The mechanisms behind cognitive interventions’ transfer and generalization effects remain unclear [19].

### Working memory and emotion self-regulation

Emotions play a crucial role in the daily lives of individuals, and regulating emotions significantly impacts the quality of life. Consequently, there has been a concerted effort by researchers and practitioners to enhance humans’ emotion regulation abilities as a preventive measure against emotional disorders [34]. While developing effective intervention methods for promoting emotion regulation remains challenging [35], several promising studies have been conducted. Wadlinger and Isaacowitz [36] proposed that gaze pattern training, particularly in a dot-probe task, is a valuable technique for enhancing emotion regulation. This type of training aims to modify the attention network functions of alerting and orientation, requiring attention disengagement from negative information and redirection towards positive or neutral information [36]. Meditative practices, such as concentration meditation, mindfulness-based stress reduction, cognitive therapy, and integrative body–mind training, have been explored as methods to promote emotion regulation [21, 37]. These practices likely engage the entire attention network, including alerting, orientation, and executive control [23].

According to the Selection, Optimization, and Compensation with Emotion Regulation (SOC-ER) framework [24, 38], successful emotion regulation relies on internal resources. This framework’s frequently adopted internal resource is the ability to control Attention, known as “working memory capacity [24]. Recent studies have shown a correlation between working memory capacity, based on attention control, and emotion regulation ability, particularly relying on the executive function processes in working memory [27, 28, 38]. Individuals with higher working memory capacity have been reported to be more successful in down-regulation tasks and experience fewer emotional responses [28]. Furthermore, a positive correlation has been observed between individual differences in reappraisal ability and working memory capacity [29]. The relationship between working memory and emotion regulation is likely mediated by attention control, with working memory being a universal processing ability and its domain-general aspect being attention control [30, 31].

Working memory capacity is crucial for processing target-related information and eliminating interference from distractions [28]. Attention control, encompassing alerting, orientation, and executive control functions, is integral to working memory capacity [32]. Individuals with attention control deficits may face challenges in allocating attention resources, leading to susceptibility to negative emotional information and difficulties in emotion regulation [33]. Negative emotional stimuli, similar to distractions, can interfere with cognitive tasks for individuals with low attention control ability [39, 40]. The present study hypothesized that improving working memory capacity through training could enhance attention control ability, subsequently improving emotion regulation ability. Attention control was considered a shared component and bridge between working memory and emotion regulation [41]. The study utilized a running memory task and the attentional network test (ANT) to enhance working memory and evaluate changes in attention control, respectively. Additionally, changes in emotion regulation were measured, and correlations were calculated to assess the relationship between ANT components and emotion regulation outcomes. In related studies [42–55], researchers often use specific emotional situations and experimental manipulations to assess participants’ emotion regulation tasks and analyze changes in emotion regulation ability based on various indices, such as electrocardiogram, electroencephalography, and behavioral performance results.

## Research method

To achieve the objectives of the study, we employed a quasi-experimental research approach, incorporating pretest-posttest and a follow-up test with repeated measures. The study focused on 120 female students enrolled in the third and fourth grades at an elementary school in Changchun, Jilin, China. Specifically, 30 students from the third and fourth grades, selected from a psychological center in Changchun, participated in the study. Inclusion criteria included obtaining conscious consent and demonstrating proficiency in reading and writing, while exclusion criteria encompassed a lack of willingness to continue participation and attendance in neurofeedback therapy sessions. In alignment with ethical principles, post-research educational sessions were provided for the control group, and participants were assured of confidentiality. The analysis of the data employed t-tests for independent samples using SPSS software version 23.

### Instruments

In this study, three instruments were employed to gather data.

- ***The SNAP-IV questionnaire***

The SNAP-IV in Children Displaying ADHD Symptoms [19] comprises 18 items categorized into two factors: inattention and hyperactivity/impulsivity. Reliability assessment was conducted using Cronbach’s alpha. The reliability of the questionnaire and its subscales, which was estimated through Cronbach’s alpha, exceeded 0.82.

b) ***Conners Comprehensive Behavior Rating Scale***

The second scale was the Conners Comprehensive Behavior Rating Scale [56], a questionnaire targeting behavioral, social, and academic aspects in children aged 6–18 years, aiding in the diagnosis of attention deficit hyperactivity disorder (ADHD). This scale, widely utilized by parents, encompasses 21 questions assessing ADHD symptoms, including attention deficits, hyperactivity or impulsivity, sleep difficulties, social challenges, and emotional experiences. Completed by mothers, it features four subscales: Oppositional, Cognitive Problems – Inattention, Hyperactivity, and the ADHD Index. Cronbach’s alpha was used for estimating the reliability of the test, and the reliability index was 0.85.

c) ***Cognitive Emotion Regulation Questionnaire***

Finally, the Cognitive Emotion Regulation Questionnaire, developed by Garnefski and Kraaij [57], was employed to evaluate participants’ cognitive emotion regulation. Comprising 36 items on a 5-point Likert scale, this questionnaire includes nine subscales, five focusing on adaptive cognitive emotion strategies and four on non-adaptive strategies. The reliability of the scale was estimated using Cronbach’s alpha and the obtained alpha exceeded 0.89, which is acceptable.

### Software for memory training

Cogmed stands as a working memory training software program commonly utilized as a supplementary intervention for ADHD. Working memory is vital in helping individuals remember instructions, solve problems, control impulses, and maintain focused Attention. Cogmed is designed to enhance the working memory capabilities of those facing challenges in retaining information or remembering tasks. Cogmed operates as a computer program comprising 25 online training sessions, each lasting 10 to 45 minutes. Users are recommended to complete five sessions per week in a setting of their choice, at home, school, work, or any other comfortable location.

Initially, a Cogmed provider interviews each patient to assess the potential benefits of the training. Subsequently, a Cogmed coach facilitates the first online session and maintains weekly calls to discuss the patient’s progress, enhance motivation, and provide feedback. Cogmed coaches and trainees can review the results of each session online. During each session, the logged-in participant engages in exercises resembling video games. These exercises include recalling and repeating the sequence in which a panel of lights or a field of asteroids is illuminated. The system responds to correct and incorrect answers, adjusting the difficulty level to challenge the limits of working memory for accurate choices and reducing difficulty for incorrect responses to prevent frustration. The exercises evaluate both visual and verbal working memory, and players typically complete around eight different exercises in each session. Upon completion of the training, the coach offers feedback during a concluding session and conducts a follow-up after 6 months to evaluate the achieved results.

### Procedure

In the initial stage of the study, participants were identified based on specific inclusion criteria, research objectives, and ethical considerations. The research purpose and participant education methods were then clarified, addressing any queries they might have had. Consent was obtained in writing from participants, their parents, and center officials. Before the commencement of emotional working memory training sessions, participants were required to complete three questionnaires: the Cognitive Emotion Regulation Questionnaire, SNAP-IV (which includes inattention and hyperactivity/impulsivity factors), and the Conners Comprehensive Behavior Rating Scale. Subsequently, participants underwent the training sessions, and they filled out the questionnaires again before each session.

Before each working memory training session, participants were given written test instructions, and researchers verbally explained the training software to ensure better comprehension. The training sessions, conducted over 25 consecutive days (excluding Saturdays and Sundays), ranged from 10 to 45 minutes each. After completing the 25 sessions, participants once again filled out the questionnaires. To evaluate the lasting effects of the training, participants retook the questionnaires without any additional training or intervention for a period of 2 months. Following the collection of data, SPSS software version 25 was utilized for analysis, employing statistical tests such as repeated measures analysis of variance (ANOVA) and the Bonferroni follow-up test.

## Findings

### Research question 1

The first research question investigated the effect of working memory training on the attention deficit of children with ADHD. Table 1 shows the Mean and SD of the participants on pre-test, post-test, and follow-up tests.

**Table 1:** Mean and Standard Deviation of Pre-test, Post-test, and follow-up Scores for Children’s Attention Deficit

Variable / Time	Pre-test (Mean ± SD)	Post-test (Mean ± SD)	Follow-up (Mean ± SD)
Attention deficit	73 ± 15.1	56 ± 14.61	55 ± 16.14

As shown in Table 1, the means of deficit attention changed across the pre-intervention, post-intervention, and follow-up stages compared to before the intervention. The analysis utilized repeated measures analysis of variance (ANOVA). In this analysis, deficit attention was entered into the model as a separate dependent variable measured at three-time points. However, the test assumptions were also examined before conducting the repeated measures ANOVA. The results of Mauchly’s sphericity test, assessing the assumption of homogeneity of variance-covariance matrices deficit attention, indicated that the assumption was met (*p* < 0.15). Therefore, no degrees of freedom adjustment is necessary for interpreting the F-test. Results are presented in Table 2.

**Table 2:** Repeated measures ANOVA for the children’s deficit attention

	Sum of Squares	df	Mean Square	F	P	Eta
Attention	32.92	2	16.40	4.56	0.001	0.62

The analysis of variance (ANOVA) was conducted to assess the impact of the training on the children’s attention. The results revealed a significant effect of Attention on the outcome, as evidenced by a substantial F-statistic of 4.56 (*p* = 0.001). The Eta value of 0.62 further emphasizes the practical significance of this relationship, suggesting that approximately 62% of the variability in the dependent variable can be explained by variations in the Attention variable. This substantial effect size underscores the importance of the Attention factor in influencing the observed outcomes. The mentioned differences were examined through Bonferroni follow-up tests, and the results of these tests are presented in Table 3. Results showed that the difference between the pre-test, post-test, and follow-up test was significant, favoring the post-test. However, the difference between the post-test and follow-up test was not significant.

**Table 3:** Bonferroni test for comparing deficit attention in pre-test, post-test, and follow-up tests

Dependent Variable	(I)	(J)	Mean Difference	p.
Attention	pretest	Posttest	17	.001
		Follow up	18	.001
	Post-test	Follow up	1	0.58

### Research question 2

The second research question investigated the effect of WM on adaptive cognitive emotion regulation. As shown in Table 4, the means of the cognitive emotion regulation subscales changed across the pre-intervention, post-intervention, and follow-up stages compared to before the intervention.

**Table 4:** Mean and Standard Deviation of Pre-test, Post-test, and follow-up Scores for Children’s adaptive cognitive emotion regulation

Variable / Time	Pre-test (Mean ± SD)	Post-test (Mean ± SD)	Follow-up (Mean ± SD)
Positive Refocusing	10.80 ± 3.20	12.80 ± 2.60	12.56 ± 2.23
Refocus on Planning	7.56 ± 1.24	10.23 ± 2.25	10.25 ± 2.31
Positive Reappraisal	8.70 ± 1.92	10.25 ± 2.30	10.36 ± 2.32
Putting Things into Perspective	7.80 ± 3.13	9.90 ± 2.23	10.12 ± 2.56
Acceptance	8.20 ± 2.23	10.10 ± 3.20	9.95 ± 2.21

As shown in Table 4, the means of all dimensions changed across the pre-intervention, post-intervention, and follow-up stages compared to before the intervention. The analysis utilized repeated measures analysis of variance (ANOVA). In this analysis, the subscales were entered into the model as a separate dependent variable measured at three-time points. However, the test assumptions were also examined before conducting the repeated measures ANOVA. The results of Mauchly’s sphericity test, assessing the assumption of homogeneity of variance-covariance matrices deficit attention, indicated that the assumption was met (*p* < 0.15). Therefore, no degrees of freedom adjustment is necessary for interpreting the F-test. Results are presented in Table 5.

**Table 5:** Repeated measures ANOVA for adaptive cognitive emotion regulation sub-scales

	Sum of Squares	df	Mean Square	F	P	Eta
Positive Refocusing	32.92	2	16.40	4.56	0.001	0.62
Refocus on Planning	62.53	2	31.23	5.66	0.001	0.69
Positive Reappraisal	27.73	2	13.86	6.23	0.001	0.68
Putting Things into Perspective	27.63	2	13.56	5.26	0.001	0.59
Acceptance	32.56	2	12.25	4.23	0.001	0.42

The analysis of various cognitive regulation strategies reveals significant effects on the dependent variable. Positive Refocusing, Refocus on Planning, Positive Reappraisal, Putting Things into Perspective, and Acceptance all demonstrate substantial impacts, as indicated by their respective F-statistics of 4.56, 5.66, 6.23, 5.26, and 4.23, each associated with a *p*-value of 0.001, underscoring the statistical significance of these effects.

Examining effect sizes (Eta), Refocus on Planning stands out with a notable value of 0.69, followed closely by Positive Reappraisal at 0.68, Putting Things into Perspective at 0.59, Positive Refocusing at 0.62, and Acceptance at 0.42. These Eta values suggest varying degrees of influence, with Refocus on Planning having the most substantial impact on the dependent variable, while Acceptance shows a moderate effect. The mentioned differences were examined through Bonferroni follow-up tests, and the results of these tests are presented in Table 6. Results showed that the difference between the pre-test, post-test, and follow-up test was significant, favoring the post-test. However, the difference between the post-test and follow-up test was not significant.

**Table 6:** Bonferroni test for comparing subscales of adaptive cognitive emotion regulation in pre-test, post-test, and follow-up test

Dependent Variable	(I)	(J)	Mean Difference	*p*.
Positive Refocusing	pre-test	Post-test	2	.001
		Follow up	1.76	.001
	Posttest	Follow up	0.24	0.58
Refocus on Planning	pretest	Posttest	2.67	.001
		Follow up	2.70	.001
	Posttest	Follow up	0.03	0.58
Positive Reappraisal	pre-test	Post-test	2.69	.001
		Follow up	2.78	.001
	Posttest	Follow up	0.09	0.58
Putting Things into Perspective	pre-test	Post-test	2.10	.001
		Follow up	2.22	.001
	Posttest	Follow up	0.12	0.58
Acceptance	pre-test	Post-test	1.90	.001
		Follow up	1.75	.001
	Posttest	Follow up	0.15	0.58

### Research question 3

The third research question investigated the effect of WM on non-adaptive cognitive emotion regulation. As shown in Table 7, the means of the non-adaptive cognitive emotion regulation subscales changed across the pre-intervention, post-intervention, and follow-up stages compared to before the intervention.

**Table 7:** Mean and Standard Deviation of Pre-test, Post-test, and follow-up Scores for Children’s non-adaptive cognitive emotion regulation

Variable / Time	Pre-test (Mean ± SD)	Post-test (Mean ± SD)	Follow-up (Mean ± SD)
Self-blame	9.60 ± 2.1	6.00 ± 1.12	6.25 ± 2.12
Rumination	12 ± 1.11	10.20 ± 9.16	10.30 ± 2.23
Catastrophizing	9.20 ± 2.23	6 ± 1.70	6.10 ± 1.80
Blaming others	7.80 ± 3.39	6.20 ± 1.23	5.80 ± 1.30

Table 7 shows the effectiveness of WM on non-adaptive cognitive emotion regulation across various strategies over time. Participants demonstrated notable improvements in adaptive strategies, as seen in self-blame reduction from an initial mean of 9.60 (± 2.1) to 6.25 (± 2.12) at follow-up. Similarly, non-adaptive rumination, catastrophizing, and blaming others exhibited positive changes post-intervention, with sustained improvements during the follow-up period. Specifically, rumination decreased from 12 (± 1.11) to 10.30 (± 2.23), catastrophizing from 9.20 (± 2.23) to 6.10 (± 1.80), and blaming others from 7.80 (± 3.39) to 5.70 (± 1.30). Repeated measures ANOVA was used; the results are presented in Table 8.

**Table 8:** ANOVA test for comparing the groups’ scores on non-adaptive cognitive emotion regulation

	Sum of Squares	df	Mean Square	F	P	Eta
Self-blame	30.92	2	15.40	5.56	0.001	0.67
Rumination	60.53	2	30.23	7.69	0.001	0.70
Catastrophizing	30.56	2	15.23	8.26	0.001	0.65
Blaming others	28.63	2	14.25	9.30	0.001	0.65

As seen in Table 8, the ANOVA results for comparing group scores on non-adaptive cognitive emotion regulation strategies indicate statistically significant differences among the groups in self-blame (F = 5.56, *p* = 0.001, Eta = 0.67), rumination (F = 7.69, *p* = 0.001, Eta = 0.70), catastrophizing (F = 8.26, *p* = 0.001, Eta = 0.65), and blaming others (F = 9.30, *p* = 0.001, Eta = 0.65). These findings underscore the significance of group variations in non-adaptive cognitive strategies, with rumination demonstrating the highest effect size. It suggests that addressing these maladaptive strategies is crucial for understanding and potentially mitigating group differences in cognitive emotion regulation. The differences were examined through Bonferroni follow-up tests, and the results are presented in Table 9. Results showed that the difference between the pre-test, post-test, and follow-up test was significant, favoring the post-test. However, the difference between the post-test and follow-up test was not significant.

**Table 9:** Bonferroni test for comparing subscales of non-adaptive cognitive emotion regulation in pre-test, post-test, and follow-up test

Dependent Variable	(I)	(J)	Mean Difference	*p*.
Self-blame	pretest	Posttest	3.6	.001
		Follow up	3.35	.001
	Posttest	Follow up	0.25	0.69
Rumination	pretest	Posttest	1.80	.001
		Follow up	1.70	.001
	Posttest	Follow up	0.10	0.50
Catastrophizing	pre-test	Post-test	3.20	.001
		Follow up	3.10	.001
	Posttest	Follow up	0.10	0.98
Blaming others	pre-test	Post-test	1.60	.001
		Follow up	2	.001
	Post-test	Follow up	0.40	0.78

## Discussion

The first research question aimed to investigate the impact of memory training on improving Attention in children with ADHD. The results of the t-test indicated that the memory training software led to an increase in attention levels. Furthermore, the findings of this study were consistent with other research, emphasizing the significant role of working memory deficits in Attention Deficit/Hyperactivity Disorder (ADHD) (Durat, Woods, 2012).

Cognitive interventions, such as memory training, have shown notably positive results, aligning with the results of other studies [1–14]. The role of executive functions, particularly inhibitory control, working memory, and planning, is crucial in individuals with ADHD. Continuous and robust deficits in these executive functions, including inhibitory response, working memory, and planning, have been reported [15, 16]. In this context, memory training software, by providing training in goal-directed skills, inhibitory response, and multi-step tasks, can enhance inhibitory response and working memory skills in these children.

Memory training focusing on auditory and visual components can effectively enhance the memory of children with ADHD. Computerized programs designed to improve various executive functions in children with ADHD are essential. The justification for the effectiveness of memory training on memory and inhibitory response in hyperactive children can be attributed to the fact that this software teaches goal-directed skills and multi-step tasks.

It is crucial to note that memory training tasks involve performing activities simultaneously, leading to cognitive interference and attentional demands. In this sense, the capacity of working memory, a limitation in an individual’s ability to retrieve information repeatedly from memory distorted and interfered with due to Attention to other cognitive activities, is considered. Therefore, memory training can improve these children’s inhibitory response and working memory [7, 9, 11].

It can be inferred that memory training and similar interventions can serve as complementary therapeutic approaches in the treatment of ADHD, potentially even as alternatives to pharmacological interventions. Due to time constraints, the limited number of sessions, and the lack of long-term follow-up in this study, future research with increased session numbers and a more extensive sample size is recommended. Additionally, exploring the differential effects of treatment based on subtypes of ADHD could provide valuable insights. The second and third research questions aimed to investigate the impact of working memory (WM) on cognitive emotion regulation strategies in children with ADHD. The results of this research indicated that working memory training led to an increase in adaptive strategies and a decrease in maladaptive strategies (such as blaming others, catastrophizing, and self-blame) in the cognitive emotion regulation of participants. The evaluation encompassed the pre-test, post-test, and follow-up stages.

The findings of this study align with previous research that demonstrated the potential of emotional working memory training to enhance individuals’ abilities in emotion regulation and emotional improvement [58–61]. These results indicate a positive relationship between working memory capacity and emotion regulation skills [62]. Consistent with theoretical foundations, individuals’ emotion regulation skills change as their working memory capacity increases. This translates to a reduction in maladaptive strategies and an increase in adaptive strategies. Individuals with higher working memory capacity tend to suppress emotional states more effectively and can adopt a non-emotional approach when faced with emotional stimuli [27, 61, 62]. Studies suggest that training working memory using emotional stimuli in adolescents facing social and emotional challenges results in improved emotional regulation abilities and reduced behavioral difficulties [7, 29, 62].

## Conclusions

In conclusion, the research has provided valuable insights into the impact of memory training on Attention and cognitive emotion regulation strategies in children with ADHD. The findings indicate that memory training, particularly software targeting working memory, can enhance attention levels in children with ADHD. These results align with existing research highlighting the significant role of working memory deficits in ADHD. Moreover, the study demonstrates the positive influence of working memory training on cognitive emotion regulation strategies, revealing an increase in adaptive strategies and a decrease in maladaptive strategies across various stages of assessment. This underscores the potential of working memory interventions to positively contribute to emotional regulation skills in children with ADHD.

The results suggest that memory training, focusing on goal-directed skills, inhibitory response, and multi-step tasks, can be a promising complementary or alternative therapeutic approach for managing ADHD symptoms, potentially serving as an adjunct to pharmacological interventions. However, it is essential to acknowledge study limitations, including time constraints and the absence of long-term follow-up, necessitating further research with extended sessions and larger sample sizes. Additionally, exploring the nuanced effects of working memory interventions based on ADHD subtypes could offer valuable insights into tailored interventions for different presentations of the disorder.

### Implications

The practical implications of the study suggest actionable strategies for supporting children with ADHD. The incorporation of memory training interventions, especially through educational software, emerges as a practical approach to enhancing attention levels and cognitive emotion regulation. This implies that educators and clinicians may find value in integrating memory training programs into classroom activities and home-based interventions, providing ongoing support for children with ADHD. Furthermore, the study emphasizes the potential of memory training as a complementary tool alongside pharmacological intervention. This dual approach, combining memory training with medication or considering memory training as an alternative for those seeking non-pharmacological interventions, aligns with practical considerations for a holistic ADHD management strategy. The findings also advocate for the sustained implementation of memory training over an extended period for enduring benefits. This highlights the practical importance of ongoing support and long-term follow-up assessments to monitor and maximize the effectiveness of memory training interventions.

Recognizing the heterogeneity within ADHD, tailoring memory training interventions to specific subtypes becomes a practical consideration. Addressing the unique cognitive and emotional challenges associated with different ADHD presentations ensures a more personalized and effective approach in practical settings. Practically, memory training activities, particularly those encompassing both auditory and visual components, could be seamlessly integrated into the school curriculum. This incorporation into daily routines offers continuous cognitive stimulation and support for working memory skills, facilitating a practical and sustainable intervention for children with ADHD.

### Implications and suggestions for further studies

While the present study sheds light on the potential benefits of Working Memory Training (WMT) for Chinese children with Attention Deficit/Hyperactivity Disorder (ADHD), certain limitations warrant consideration. Firstly, the sample size in this study was relatively modest, which might limit the generalizability of the findings. Future studies could benefit from larger and more diverse samples to enhance the external validity of the results. Additionally, the study’s duration and the number of WMT sessions were relatively short, hindering a comprehensive understanding of the long-term effects of the intervention. Extending the duration of the intervention and conducting follow-up assessments over an extended period could provide valuable insights into the sustainability of the observed improvements.

Furthermore, the lack of a control group receiving a different intervention or no intervention poses a challenge in attributing the observed changes solely to WMT. Including a well-designed control group would strengthen the internal validity of the study and allow for a more rigorous examination of the specific effects of WMT. Moreover, considering the heterogeneity within ADHD, future research could explore the differential effects of WMT on various subtypes of the disorder. Tailoring interventions to specific ADHD presentations might offer a more nuanced understanding of the intervention’s efficacy. Finally, incorporating objective measures, such as neuroimaging or physiological assessments, could provide additional objective indicators of cognitive and emotional changes associated with WMT in children with ADHD.

## Data Availability

No datasets were generated or analysed during the current study.
